# Running exercise strengthens the intervertebral disc

**DOI:** 10.1038/srep45975

**Published:** 2017-04-19

**Authors:** Daniel L. Belavý, Matthew J. Quittner, Nicola Ridgers, Yuan Ling, David Connell, Timo Rantalainen

**Affiliations:** 1 Deakin University, School of Exercise and Nutrition Sciences, Institute for Physical Activity and Nutrition, 221 Burwood Highway, Burwood, Victoria, 3125, Australia; 2 Imaging at Olympic Park, 60 Olympic Boulevard, Melbourne, Victoria, 3004, Australia; 3 Monash University, Wellington Road, Clayton, Victoria, 3168, Australia

## Abstract

There is currently no evidence that the intervertebral discs (IVDs) can respond positively to exercise in humans. Some authors have argued that IVD metabolism in humans is too slow to respond anabolically to exercise within the human lifespan. Here we show that chronic running exercise in men and women is associated with better IVD composition (hydration and proteoglycan content) and with IVD hypertrophy. Via quantitative assessment of physical activity we further find that accelerations at fast walking and slow running (2 m/s), but not high-impact tasks, lower intensity walking or static positions, correlated to positive IVD characteristics. These findings represent the first evidence in humans that exercise can be beneficial for the IVD and provide support for the notion that specific exercise protocols may improve IVD material properties in the spine. We anticipate that our findings will be a starting point to better define exercise protocols and physical activity profiles for IVD anabolism in humans.

We expect that tissues will adapt to loads placed upon them. Wolff^1^ described the theory of bone adaptation to loading. In the intervening years, evidence^2^ has been obtained as to which loading protocols are beneficial (osteogenic) for bone. For the intervertebral disc (IVD), little is known about what loading protocols are beneficial for IVD tissue and cause anabolism in humans. We have good knowledge of loading types that are more likely to damage lumbar IVD tissue in humans, such as flexion of the spine with compression^3^, torsion^4^ or to damage to the vertebral end-plate via axial compression^3^ with subsequent IVD degeneration^5^. Whilst this information can inform what activities people should avoid to preserve IVD integrity, it does not inform us on exercise or habitual physical activity to “strengthen” the IVD. Furthermore, data^6^ on turnover rates in the IVD, lead to the assumption that positive adaptation in the mature IVD is unlikely to occur during the normal human lifespan.

Currently we rely on data from animal, IVD cell and IVD tissue models to suggest what kind of loading might be beneficial for the human lumbar IVD. These models suggest^7^ that a “likely anabolic loading window” for the IVD exists: dynamic loading of 0.2–0.8 MPa, generating intra-discal pressures of approximately 0.3–1.2 MPa, at 0.1 to 1 Hz for approximately eight hours a day. Given human data on intra-discal pressures in different activities^8^, this could^9^ be extrapolated to suggest that walking or running exercise is likely anabolic for the IVD. Quadrupedal treadmill running exercise in rodents^10^,^11^ can have a positive impact on the rodent IVD. However, directly applying loading thresholds and protocols from animal models to humans is problematic^12^ and there is no evidence yet^9^ of a beneficial effect of exercise on the IVD in humans. We aimed to determine whether beneficial effects on the IVD of exercise can be seen in humans and what loading patterns this might entail.

Our hypothesis was that people who perform regular upright running activity will show better IVD tissue quality, as shown by higher T2-times^13^ in their lumbar IVDs, than people who are healthy with no history of spinal disease, but otherwise not physically active. We also hypothesised that there would be a dose-response effect of different volumes of running. Furthermore, to better understand what types of physical activity are likely beneficial for IVD, we explored the relationship between habitual physical activity, as measured by objective accelerometry, and IVD characteristics. To reduce the confounding influence of normal aging on our findings, and given evidence^9^ that IVD maturation is still in process in the third decade of life, we included women and men aged 25–35 years. It is also not clear how long is required before the IVD might show a measurable adaptation to exercise, and we therefore recruited only people with a minimum of 5 years history at their current physical activity level: either no sport (referents), 20–40 km per week running (joggers), or 50 + km per week running (long-distance runners).

## Results

**Figure 1:**
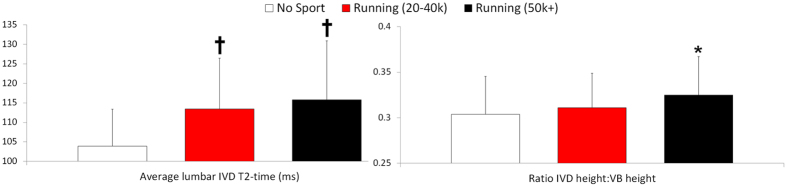
Runners have more hydrated (left) and hypertrophied (right) lumbar IVDs. Values are mean(SD) averaged across all lumbar discs. Left panel: Higher T2-times indicate^13^ better IVD hydration and glycosaminoglycan content. Right panel: IVD height relative to vertebral body height. *p < 0.05; ^†^p < 0.01 and indicate significance of difference to the non-sporting group.

**Figure 2:**
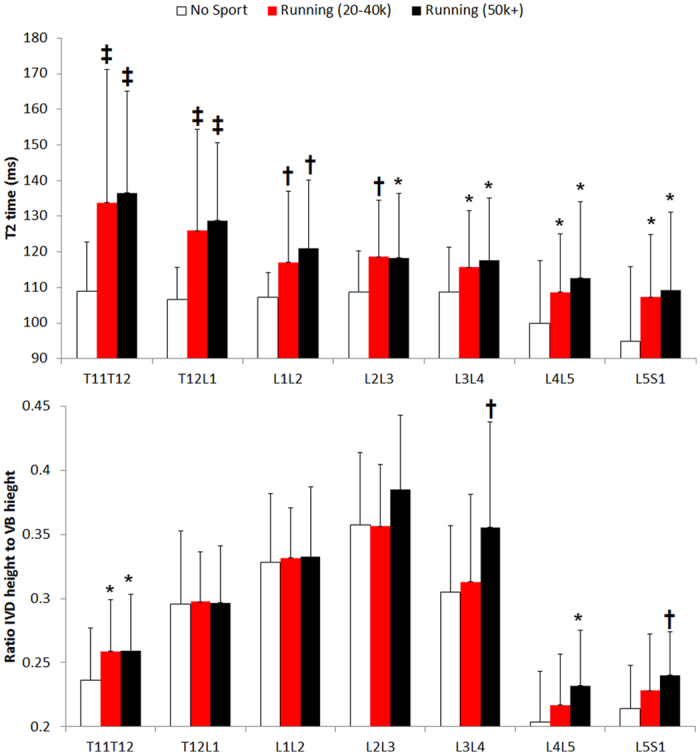
Effect of running is also present at lowest lumbar vertebral levels: T2-time (top) and IVD height relative to vertebral body height (bottom). Values are mean(SD) at each vertebral level. *p < 0.05; ^†^p < 0.01; ^‡^p < 0.001 and indicate significance of difference to the non-sporting group. VB: vertebral body.

**Figure 3:**
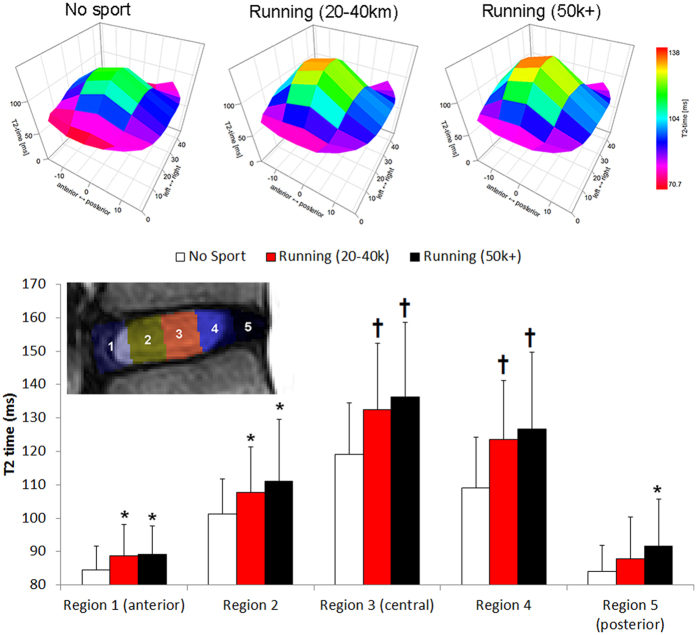
The impact of running on the disc is strongest in the nucleus. Top: 3D plot of mean T2-time across entire IVD volume. Bottom: At the mid-line (sagittal) portion of the IVD the impact of running can be seen to be greatest in the central, nuclear, portion of the IVD. *p < 0.05; ^†^p < 0.01 versus non-sporting group. Greater T2-times indicate^13^ better IVD hydration and glycosaminoglycan content.

Long-distance runners (Table 1) and joggers showed significantly higher (+11.4% and +9.2% respectively) lumbar IVD T2-times than the non-athletic individuals (Fig. 1). This effect was also present at all individual vertebral levels T11/T12 to L5/S1 (Fig. 2; upper panel). The effect of running on T2-time was strongest in the IVD nucleus (Fig. 3; +11% in joggers and +15% in long-distance runners in the central nuclear region versus +5% to +6% respectively in the anterior annulus and +5% and +9% respectively in the posterior annulus). The height of the IVD relative to that of the vertebral body, an indicator of IVD hypertrophy, was greater in the long-distance runners (Fig. 1). When examining individual vertebral levels (Fig. 2; lower panel), this effect was present at the lower lumbar vertebral levels L3/L4 to L5/S1. Lumbar muscle size did not differ between groups (Table 1). The effect of running exercise was consistent for both genders, with the gender × group interaction not reaching significance (*p* > 0.18). The long-distance group typically showed greater differences in IVD parameters to the non-sporting group than the jogging group. However, there were no statistically significant differences between the two running groups.

**Figure 4:**
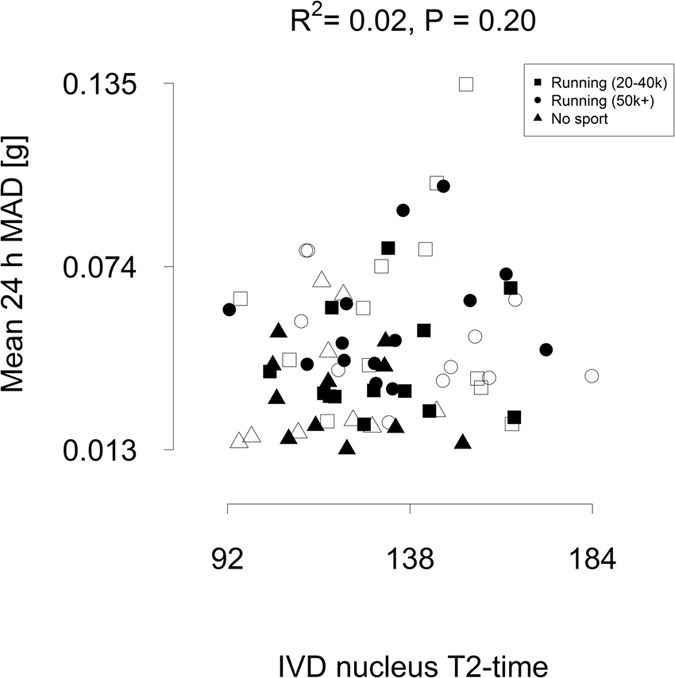
Total physical activity levels were unrelated to IVD characteristics. Empty symbols = males, filled symbols = females. Individual values for each subject shown. There was no correlation between total physical activity levels and T2-time in the intervertebral disc (IVD) nuclear region.

**Figure 5:**
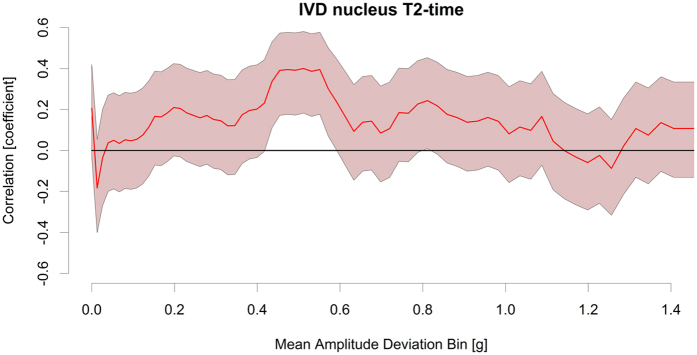
Specific loading levels impact the IVD nucleus T2-time. Values are mean(95% CI) correlation between IVD nucleus T2-time and count data in each mean amplitude deviation bin. The strongest association to higher IVD T2-times were seen between 0.44 and 0.59 g. This corresponds to fast walking and slow jogging (see Results and Supplementary Table 1).

Total physical activity levels, as measured by objective accelerometry, were not related to IVD characteristics (Fig. 4). Rather, IVD nucleus T2-time was most strongly associated with accelerations in the range 0.44 and 0.59 *g* mean amplitude deviation (MAD; Fig. 5). Additional accelerometry data collected under different exercise conditions in ten individuals (Supplementary Table 1) showed that ambulation at 2 m/s fell inside this 0.44 and 0.59 *g* MAD range. Walking at 1.5 m/s or slower fell below this range and running at 2.5 m/s or faster and jumping were above this range.

## Discussion

Before the first interventional studies to define exercise regimes for improving bone characteristics were performed in the 1990s, an important step was the finding of differences in bone density between different athletic populations^14^. This built on prior animal studies and helped to show that exercise may well result in anabolic adaptation of bone. In this vein, the current study builds on prior work in animal, cell and tissue explant models^7^ and provides the first ever cross-sectional evidence in humans that exercise may well favourably impact the IVD.

Our main finding was that long-distance runners and joggers showed better hydration and glycosaminoglycan levels (higher lumbar IVD T2-times^13^) than the non-athletic individuals. This is consistent with findings of an anabolic response in the IVD in quadrupedal animals^10^,^11^ to running. The finding is also consistent with the notion, developed on the basis of cell, tissue explant and animal models^7^,^15^, of a “likely anabolic window” for IVD loading. The effect of running in humans on IVD composition was most obvious in the IVD nucleus where intra-discal pressure increases with applied axial load are constrained by the ring-formed annular fibres^16^.

Beyond the compositional differences, there was evidence of IVD hypertrophy in the long-distance runners. The height of the IVD relative to that of the vertebral body, which serves as an internal control for body size, was greater in the long-distance runners. This extends on findings^17^ from monozygotic twins that IVDs were marginally, but not significantly, larger in those twins that were at least 8 kg heavier than their twin pair and presumably experienced greater habitual spinal loading. Hypertrophy of the IVD may well be an adaptation to habitual loading in runners. Similar to hypertrophic responses seen in muscle due to resistance training^18^, this suggests that tissue adaptation will occur in the IVD with exercise. Overall our findings provide support for the hypothesis that an adaptive, anabolic and hypertrophic response is possible in the human IVD with exercise.

The current study also provides some guidance on what kinds of loading protocols may be better for the IVD. To understand what kinds of physical activity in humans might be the drivers of an anabolic response in the IVD, we examined the physical activity patterns of our collective via objective accelerometry. Total physical activity levels were not related to positive adaptations in the IVD, rather accelerations in a specific range. The strongest association to higher IVD T2-times were seen between 0.44 and 0.59 *g* mean amplitude deviation. This fits with the idea from animal, tissue and cell models^7^ of a “likely anabolic window” for the IVD. To better understand what kinds of activities generate these acceleration magnitudes, we collected additional accelerometry data under different conditions. Walking or slow running at 2 m/s fell inside this range with slower walking falling below this range. Fast running and high-impact jumping activities were above this range. This is in line with the notion that high-impact loading is considered^7^,^19^ to be detrimental to the IVD and vertebral end-plate. Dynamic IVD loading of 0.2–0.8 MPa, generating intra-discal pressures of approximately 0.3–1.2 MPa, is thought^7^ to be an optimal loading magnitude for the IVD. Based upon data on *in vivo* intradiscal pressures^8^, activities such as walking and running, but not lifting a 20 kg load or lying down, fall into this loading magnitude window. This corresponds well to our observations here of the impact of exercise in athletes. We also noted that sedentary activities were unrelated to IVD characteristics. In light of prior work, the results of our study suggest that, in comparison to other locomotor activity, fast walking or slow running may provide the strongest anabolic stimulus for adaptation in the IVD in humans.

Whilst the long-distance running group showed consistently better IVD properties than the jogging group, there were no statistically significant differences between the two. Also, there was no relationship between the IVD characteristics and physical activity in the 0.7 to 0.9 g MAD range where the physical activity associated with running was most evident (Supplementary Figure 1). This indicates a ceiling effect of exercise for the IVD was approached for both volume of upright axial spine loading and intensity. A ceiling effect in relation to exercise has been observed for muscle hypertrophy^20^ and bone adaptation to exercise^21^. It is possible that high volume or intensity running is not required for a beneficial adaptation in the IVD.

In the wider population, it is the lower lumbar IVDs that are most commonly affected by degeneration^22^. Furthermore, repetitive loading of the spine is considered^23^ to be a contributory factor to the development of IVD degeneration. Despite repetitive loading of the spine during running, the exercise groups of the current study did not show any detrimental effects at these lower lumbar segments. In contrast, the long-distance runners and joggers showed evidence of better IVD hydration and glycosaminoglycan content in the lower lumbar spine than those that did not perform sport. Furthermore, the evidence for IVD hypertrophy subsequent to habitual running was strongest at the lower lumbar levels. Our data show that repetitive axial loading of the spine under body weight during running in otherwise healthy people may well be beneficial for the lower lumbar IVDs.

It is important to consider some of the limitations of the current study. We performed a cross-sectional study as a first step to examine whether certain types of exercise might be beneficial for the IVD in humans. In this design it is not possible to completely rule out other confounding factors. We showed, for example, that lumbar muscle size was similar in all groups and this indicates that muscle adaptation *per se* is not the likely cause of differences in IVD characteristics in our populations. However, we cannot rule out other factors such as differences in muscle function, differences in nutrition, systemic hormonal (e.g. growth factors, cytokines, stress hormones) differences, or other indirect effects. To definitively determine that the mechanical loading from specific exercise forms result in positive adaptations in the IVD, to determine mechanisms of action, and delineate exercise guidelines for strengthening the IVD, randomised controlled exercise trials are necessary.

That an optimal loading level and pattern for the IVD exists makes sense from a tissue homeostatis perspective. Given that tissues such as bone^21^ and muscle^18^ have optimal loading conditions for an anabolic response, for the IVD we should expect it to be no different. Our findings support the perspective that, when loaded appropriately, anabolic IVD adaptation can occur in humans and that this can occur on time frame for it to be meaningful within the human lifespan.

Evidence that the IVD will respond anabolically in humans to certain types of loading may have public health implications. Spinal pain consistently presents one of the greatest costs to developed societies for disability and lost productivity, including when increased death rates in other diseases are accounted for^24^. IVD degeneration and herniation is one important contributing factor to spinal pain. Similar to understanding the impact of specific exercise in other disease constellations, for example in type II diabetes^25^, knowing that the IVD can respond to certain kinds of loading, and understanding what kinds of loading are optimal, will result in better exercise guidelines for the prevention and management of spinal pain.

## Methods

### Ethical approval and subjects

The study was approved by the Deakin University Faculty of Health, Medicine, Nursing & Behavioural Sciences human ethics advisory group. All subjects gave their informed written consent prior to participation in the study. All methods were performed in accordance with the relevant guidelines and regulations. To reduce the impact of normal ageing on the findings, only individuals aged 25 to 35 years of age were included. Exclusion criteria included current spinal pain, history of spinal surgery, history of traumatic injury to the spine, known scoliosis for which prior medical consultation was sought, current or prior smoker, known claustrophobia and possible pregnancy. We recruited three groups of people with distinct loading histories: joggers (20–40 km per week), long-distance runners (50+ km per week) and non-sporting referents. To be included in the jogging group, subjects needed to have been running 20–40 km per week for a minimum of the last 5 years and perform no other sport or exercise type more than once per week. Long-distance runners were required to have performed at least 50 km per week for a minimum of the last 5 years and, with the exception of resistance exercise for muscle hypertrophy (muscle hypertrophy training is a common training component of long-distance runners), no other sport or exercise type more than once per week. Included in the “no-sport” group were individuals who performed no regular sport or exercise in the last five years, currently performed less than 150 minutes of moderate activity (defined as activity that “causes an individual to breathe harder than normal”) per week^26^, and walked less than 15 min to or from their place of work. A total of 79 participants were included in the study (Table 1).

### Testing and scanning protocol

Participants were instructed not to perform any exercise on the day of their scan. Due to normal diurnal variation in IVD water content, all testing was performed after midday. Upon arriving at the radiology facility, participants were required to sit for a minimum of 20 minutes prior to entering the scanner with participants sitting for a mean(SD) of 44(16) minutes which did not differ between groups (p > 0.19). During this time participants completed questionnaires detailing their gender, type of physical activity, body height, and average sitting duration Monday to Friday. The runners also reported distance run per week, time run per week and number of years of participation.

To quantify IVD T2-time and morphology, a spin-echo multi-echo sequences on a 3T Phillips Ingenia scanner (Amsterdam, Netherlands) was used with spinal coils to collect images at 8 echo times (15.75, 36.75, 57.75, 78.75, 99.75, 120.75, 141.75 and 162.75 ms) from 13 sagittal anatomical slices each (thickness 3 mm; interslice distance: 1.5 mm; repetition time: 2000 ms, field of view: 281 × 281 mm, image resolution: 0.366 mm per pixel) encompassing the entire lower spine from left to right. For radiological categorisation of IVD degeneration (Pfirrmann grade), a sagittal plane T2-weighted sequence (15 slices, slice thickness: 3 mm, interslice distance: 1.5 mm, repetition time: 2600 ms, echo time: 70 ms, field of view: 357 mm × 357 mm, resolution: 0.532 mm per pixel) was taken. To quantify muscle morphology, a paraxial T1-weighted scan (repetition time: 800 ms, echo time: 9 ms, slice thickness: 4 mm, interslice distance: 2 mm, field of view: 258.68 × 258.68 mm, image resolution: 0.270 mm per pixel) with five groups of three slices each positioned at each vertebral body L1 to L5 and oriented to the vertebral end-plates was performed. Data were exported for further offline processing.

After scanning, subjects were given a hip-mounted ActiGraph model GT3X+ (Pensacola, FL). Participants were instructed to wear the ActiGraph during all waking hours except during water-based activities (e.g. swimming and bathing) for eight consecutive days. Acceleration data were collected at 100 Hz with a ±6 g range and 12 bit analog to digital conversion.

### Offline image processing and analysis

To ensure blinding of the examiner to offline image measurements, each subject was assigned a random numeric code (obtained from www.random.org). A radiologist determined the Pfirrmann grade of each lumbar IVD (Table 1) on sagittal T2-weighted images. Seven individuals had a supernumerary lumbar vertebral segment and the additional IVD (designated L6/S1) in these subjects was not included in analyses.

ImageJ 1.38x (http://rsb.info.nih.gov/ij/) was used to perform all quantitative MRI measures. In the sagittal spin-echo multi-echo images every IVD from T11/T12 to L5/S1 was measured. After segmenting the IVD, a custom written ImageJ plugin (“ROI Analyzer”; https://github.com/tjrantal/RoiAnalyzer) was used to rotate the region of interest to the horizontal and measure area, height, width and signal intensity of the IVD in its entirety as well as in five subregions from the anterior to posterior aspect of the disc. T2-time was calculated via linear fit to the natural logarithm of the image intensity in each of the eight MR echos. Similar parameters were generated for each of the five disc subregions and interpolated across the width of the IVD to generate the 3D plots presented in Fig. 3. Volume of each IVD in each subject was calculated by linear interpolation of the area data from all slices. The image number where each spinous process was best visible was noted. The vertebral body was also segmented and vertebral body height measured in a similar fashion in order to calculate the ratio of IVD height to vertebral body height, as a normalised indicator of IVD hypertrophy. The data from each lumbar IVD were also averaged. With the exception of IVD volume (Table 1), data averaged from the three images around the spinous process were used in analysis.

In each of the paraxial T1-weighted images, area of the lumbar multifidus, erector spinae, psoas and quadratus lumborum were measured bilaterally from L1 to L5 as in prior work^27^. The muscle area averaged from all lumbar levels was used in analysis.

### 3D accelerometry analysis

Once participants returned the Actigraph, raw data were downloaded from the device and analysed with a custom-written Matlab script (R2015b, Mathworks, Inc., Natick, MA, USA). Resultant acceleration was calculated from the 3-dimensional data, and used in all further analyses. No smoothing was applied on the recorded signal.

Mean amplitude deviation (
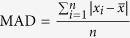
, where n = number of data points in an epoch, i = data point index, x = resultant acceleration, 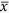 = mean of the epoch, and vertical bars signify taking the absolute value)^28^ was calculated from the resultant acceleration in non-overlapping 5 s epochs in 24 h segments starting at 6 am local time for all of the recorded days. The 5 s epoch mean MADs were divided into 98 logarithmically equidistant bins (=histogram) from 0 g to 2.5 g. Non-wear time was defined as any hours with standard deviation less than 0.024 g, and any days with less than 10 h of total wear-time were excluded from analysis. Data from individuals with ≥3 days of sufficient wear-time were included. 70 individuals completed the Actigraph data collection and were included in the analysis. Two individuals from the no-sport group, six from the jogging group and one from the long-distance running group did not complete this data collection. On average, 7.6 days of complete data were available per participant (similar across all three groups, *p* = 0.9). The mean of all included days is reported.

### Additional sub-study: which activities generate what accelerations?

To relate MAD to physical activities, 10 individuals performed a graded treadmill test at speeds of 0.5 m/s, 1 m/s, 1.5 m/s, 2 m/s, 2.5 m/s, 3.0 m/s and 3.5 m/s whilst wearing a hip-mounted ActiGraph. At each speed, 70 seconds of data were collected and the data from the 11 second onwards was used for further analysis. Participants also performed 10 consecutive jumps maintaining the knee and hip at near full extension (similar to hopping on one leg, but bilaterally).

### Statistical analyses

An alpha-level of 0.05 was taken for statistical significance. For continuous variables, T-tests were performed comparing the long-distance and jogging groups to the non-sport referent group. Primary analysis evaluated IVD T2-time (composition measure) and IVD height to vertebral body height ratio (hypertrophy measure) averaged across all lumbar levels. Data from individual vertebral levels were also evaluated. Difference in response between males and females was evaluated via two-way analysis of variance for ‘group’ and ‘gender’. For comparing physical activity to IVD characteristics, the correlation and 95% confidence interval was calculated between number of counts for each subject in the MAD bin and the average lumbar IVD T2-time in the nucleus (central subregion). The mean and 95% confidence interval of the MADs in each of the physical activities of the sub-study were calculated. The “R” statistical environment (version 2.10.1, www.r-project.org) was used for all analyses.

## Additional Information

**How to cite this article:** Belavý, D. L. *et al*. Running exercise strengthens the intervertebral disc. *Sci. Rep.* **7**, 45975; doi: 10.1038/srep45975 (2017).

**Publisher's note:** Springer Nature remains neutral with regard to jurisdictional claims in published maps and institutional affiliations.

## Supplementary Material

Supplementary Table and Figure

## Figures and Tables

**Table 1:** Participant characteristics, intervertebral disc characteristics and lumbar muscle morphology.

	No sport	Running (20–40 km)	Running (50 k + )
Number of males (of total N)	11 of 24	13 of 30	11 of 25
Body mass (kg)	73.9(17.8)	68.2(11.1)	63.5(10.2)*
Age (yrs)	29.3(3.7)	30.2(3.2)	30.1(3.9)
Height (cm)	173.2(8.7)	173.6(9.7)	170.3(9.2)
Weekday sitting time (hrs)	9.7(2.1)	6.3(2.8)‡	6.4(2.8)‡
Exercise participation (yrs)	-	8.8(4.2)	7.6(3.9)
Exercise participation (hrs/wk)	-	4.9(2.2)	8.6(4.3)
Exercise distance (km/wk)	-	28.0(6.7)	66.6(19.5)
IVD volume (cm^3^)	9.5(2.3)	10.1(3.4)	10.1(3.3)
IVD average area (mm^2^)	250.2(41.2)	263.6(60.8)	263.0(58.9)
IVD height (mm)	7.1(0.7)	7.3(1.1)	7.5(1.0)
IVD anteroposterior width (mm)	25.6(2.4)	26.4(3.5)	25.9(3.4)
Intervertebral distance (mm)	34.2(2.0)	34.1(2.9)	34.0(2.8)
Pfirrmann grade	2.3(0.39)	2.2(0.39)	2.1(0.39)
Erector spinae size (cm^2^)	14.3(3.3)	14.4(5.0)	14.1(4.8)
Lumbar multifidus size (cm^2^)	4.7(1.1)	4.4(1.6)	4.1(1.6)
Psoas size (cm^2^)	9.1(2.8)	10.1(4.2)	9.6(4.0)
Quadratus lumborum size (cm^2^)	3.5(1.1)	3.4(1.7)	3.1(1.6)

Values of continuous variables are mean(SD). *p < 0.05; ^†^p < 0.01; ^‡^p < 0.001 and indicate significance of difference to the non-sporting group. The proportion of females:males did not differ across groups (χ^2^ = 0.01, p = 0.99). Three participants were of Asian descent (one female 20–40 km runner, one male 20–40 km runner, one male 50 + km runner) and the remaining participants were Caucasian. Pfirrmann grade was averaged from all lumbar discs. Areas of erector spinae, multifidus, psoas and quadratus lumborum muscles were averaged from left and right sides of the body and all lumbar levels.
