# Supplementary material for: Running exercise strengthens the intervertebral disc
Source: Sci Rep. 2017 Apr 19;7:45975. doi: 10.1038/srep45975 (PMC5396190; doi:10.1038/srep45975)
Supplement: Supplementary Table and Figure [file srep45975-s1.doc]

Supplementary Material to: **Running exercise strengthens the intervertebral disc**

Authors: **Daniel L. Belavý, Matthew Quittner, Nicola Ridgers, Yuan Ling, David Connell, Timo Rantalainen**

**Supplementary Table 1**: Acceleration mean amplitude deviation (MAD) in different activities

| **Activity** | **Velocity (*m/s*)** | **Acceleration MAD (*g*)** |
| --- | --- | --- |
|  |  |  |
| Treadmill gait | 0.5 | 0.066(0.064-0.067) |
| Treadmill gait | 1 | 0.144(0.140-0.147) |
| Treadmill gait | 1.5 | 0.272(0.265-0.278) |
| Treadmill gait | 2 | 0.48(0.46-0.51) |
| Treadmill gait | 2.5 | 0.77(0.75-0.78) |
| Treadmill gait | 3 | 0.82(0.81-0.84) |
| Treadmill gait | 3.5 | 0.86(0.85-0.88) |
| Jumping | - | 1.26(1.24-1.27) |

Values are mean(95% confidence interval). Ten subjects were measured. Two subjects transitioned to jogging at 2m/s and the remaining eight at 2.5 m/s in the treadmill protocol.

**Supplementary Figure 1**: Profiles of physical activity at different intensities: running activities unlikely to be necessary for stimulating IVD anabolism.


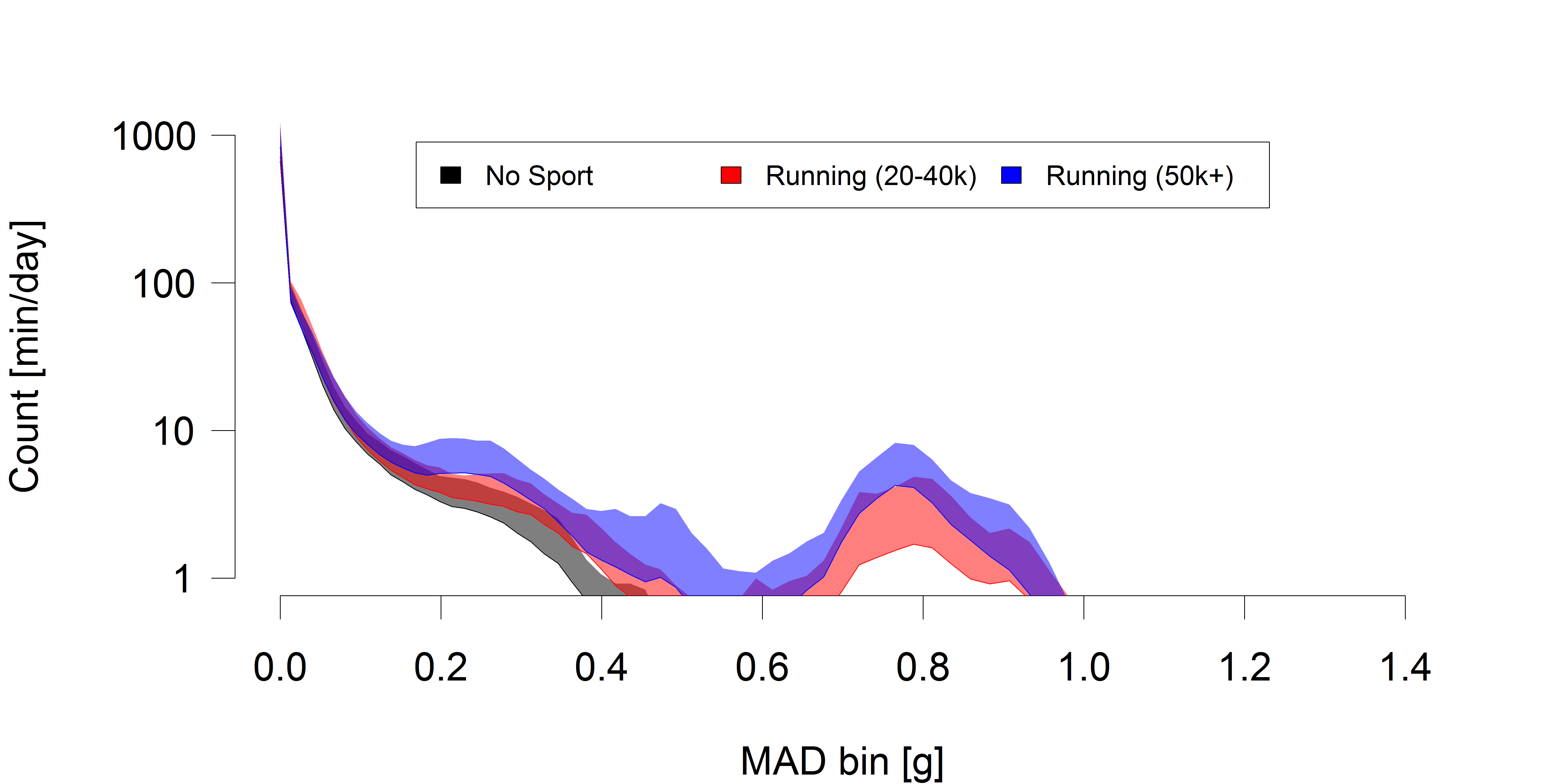


Values are 95% confidence interval (shaded) counts in each mean amplitude deviation (MAD) bin. The greatest difference between the groups in terms of physical activity was seen above 0.6*g* MAD. This was above the likely anabolic window of 0.44-0.59*g* MAD for the IVD (Figure 5), indicating that this high level of activity may not necessary to stimulate the IVD. The impact of running in the athletic groups on measured physical activity patterns can be seen most strongly in the 0.7 to 0.9*g* MAD range.
